# Hydrogen sulfide mediates athero-protection against oxidative stress via *S*-sulfhydration

**DOI:** 10.1371/journal.pone.0194176

**Published:** 2018-03-08

**Authors:** Sau Ha Cheung, James Yun Wong Lau

**Affiliations:** Department of Surgery, Prince of Wales Hospital, The Chinese University of Hong Kong, Shatin, Hong Kong; University of Louisville, UNITED STATES

## Abstract

*S*-sulfhydration is a signalling pathway of hydrogen sulfide (H_2_S), which is suggested as an anti-atherogenic molecule that may protect against atherosclerosis. The identification of *S*-sulfhydrated proteins by proteomic approach could be a major step towards understanding the mechanisms of H_2_S in response to atherosclerosis. The present study studied targeted *S*-sulfhydrated proteins using the modified biotin switch method followed by matrix-assisted laser desorption/ionisation time of flight tandem mass spectrometry identification. The results showed that H_2_S can protect against atherosclerosis by reducing body weight gain and alleviating aortic plaque formation. In addition, H_2_S treatment can increase aortic protein *S*-sulfhydration. Seventy targeted *S*-sulfhydrated aortic proteins were identified, mainly involved in metabolism, stimulus response and biological regulation, as determined by gene ontology database analysis. H_2_S also induced *S*-sulfhydration of glutathione peroxidase 1 and further reduced lipid peroxidation and increased antioxidant defence in the aorta by prompting glutathione synthesis. Our data suggest that H_2_S is a cardiovascular-protective molecule that *S*-sulfhydrates a subset of proteins that are mainly responsible for lipid metabolism and exerts its cytoprotective effects to clear free radicals and inhibit oxidative stress through cysteine *S*-sulfhydration.

## Introduction

Hydrogen sulfide (H_2_S) is one of the three molecules (along with nitric oxide and carbon monoxide) most recently identified as gasotransmitters [1]. Despite its toxicity, it is recognised as an endogenously produced gaseous signalling molecule that induces a variety of beneficial reactions in cardiovascular tissues [2]. H_2_S activity upregulates such physiological properties as vasodilation, anti-inflammation, anti-oxidation and angiogenesis [3]. In addition, accumulating evidence suggests an association between atherosclerosis and deficiency in vascular cystathionine γ-lyase (CSE), an enzyme responsible for endogenous H_2_S production. It was shown by using CSE knockout mice that CSE gene deletion led to a decrease in H_2_S production with accelerated atherosclerosis [4]. Atherosclerotic plaques could also be ameliorated by overexpression of CSE in apolipoprotein E knockout (ApoE KO) mice [5] and H_2_S supplementation by exogenous H_2_S donors such as NaHS [6] and GYY4137 [7]. H_2_S is therefore suggested as an anti-atherogenic molecule. However, the mechanism by which H_2_S reduces atherosclerosis is still not clear.

Recently, H_2_S signalling through *S*-sulfhydration has been reviewed, and *S*-sulfhydration has been recognised as a key mechanism of H_2_S physiology [8]. Similar to *S*-nitrosylation, *S*-sulfhydration can cause post-translational modification of proteins [9]. *S*-sulfhydration takes place at reactive cysteine residues in proteins and results in the conversion of an–SH group of cysteine to an–SSH group. This conversion occurs in many proteins and modulates the functions of a targeted protein [9]. For example, H_2_S was shown to protect gastric epithelial cells from ischemia-reperfusion injury by Keap1 sulfhydration and further Keap1/Nrf2 disassociation and enhancement of Nrf2 nuclear translocation, which eventually attenuated oxidative stress and protected against mucosal injury [10]. Recently, H_2_S was suggested to inhibit the development of atherosclerosis through upregulating protein *S*-nitrosylation, thereby inhibiting the proliferation and migration of vascular smooth muscle cells [11]. Cystathionine β-synthase (CBS), another H_2_S-producing enzyme, was found to regulate endothelial function via protein *S*-sulfhydration, maintaining vascular health and function [12]. All these observations suggest the potential value of investigating *S*-sulfhydration in the context of atherosclerosis.

H_2_S signalling via *S*-sulfhydration is still not fully elucidated, in that only a few proteins have been identified to be *S*-sulfhydrated by H_2_S. To investigate H_2_S signalling in atherosclerosis, meta-analysis of *S*-sulfhydrated proteins in aortic proteins is the main step to be examined. Here we explore *S*-sulfhydrated proteins in aortic tissues of ApoE KO mice on an atherogenic diet and H_2_S treatment. We detected *S*-sulfhydration by modified biotin switch assay coupled with advanced proteomic identification strategies and analysed altered *S*-sulfhydration levels of proteins. Potential targets of protein *S*-sulfhydration were identified in aortic tissue. The difference in *S*-sulfhydration levels of proteins in aortic tissue indicates the possibility of functional alteration as well as disease-related consequences.

## Materials and methods

### Animals and experimental design

Male wild-type C57BL/6J mice (WT) and ApoE KO mice were used in this study. All mice were housed in a controlled environment with free access to water and food on a 12-h light/dark cycle. WT mice were used as a reference of healthy mice without atherosclerosis. Atherosclerotic mice, ApoE KO mice were treated with or without H_2_S in the experiment. Briefly, they were fed a normal diet until 8 weeks of age and then switched to an atherogenic diet. After 4 weeks of the atherogenic diet, the ApoE KO mice were divided into two groups: vehicle control treatment with normal saline and H_2_S treatment with GYY4137 (Cayman Chemical Co., Ann Arbor, MI, USA), a novel H_2_S donor [13]. The H_2_S supplement experiment consisted of daily IP injection of GYY4137 (133 μmol/kg body weight for 4 weeks of atherogenic diet feeding) in the ApoE KO mice. Control ApoE KO mice were injected with normal saline alone. Body weights of mice were recorded weekly. Food uptake per mouse per day was calculated as follows: [total food intake per cage]/[mice per cage]/[days of food consumption]. After 4 weeks of treatment, all mice were sacrificed by using slow fill carbon dioxide asphyxiation. Non-clotted blood was collected from each mouse. Aortic tissues were excised, immediately frozen in liquid nitrogen and stored at –80°C until further processing. All animal experiments were approved by the Animal Experimentation Ethics Committee (AEEC) of the Chinese University of Hong Kong (CUHK) and in accordance with the Animals (Control of Experiments) Ordinance (Cap. 340) licensed from the Hong Kong Government Department of Health. They were performed conform the university’s regulations on Use of Experimental Animals which incorporate The International Guiding Principles for Biomedical Research Involving Animals and The Hong Kong Code of Practice for Care and Use of Animals for Experimental Purposes.

### Release of H_2_S in plasma and tissues after GYY4137 treatment

To detect H_2_S concentration *in vivo* after GYY4137 treatment, tissues (liver and kidney) and blood were collected at timed intervals (0 to 8 hours). H_2_S level in tissue homogenates was measured by zinc acetate assay as described previously with some modifications [13]. Briefly, liver and kidney were homogenized in ice-cold 100 mM potassium phosphate buffer (pH 7.4). L-cysteine (10 mM; 20 μl), pyridoxal 5’-phosphate (2 mM; 20 μl) and saline (30 μl) were added into the homogenate (430 μl). After incubation at 37°C for 30 min, zinc acetate (1% w/v, 250 μl) was injected to trap-generated H_2_S followed by trichloroacetic acid (10% w/v, 250 μl) to precipitate protein and thus stop the reaction. Subsequently, N,N-dimethyl-p-phenylenediamine sulfate (20 μM; 133 μl) in 7.2 M HCl was added followed by FeCl_3_ (30 μM; 133 μl) in 1.2 M HCl. After 15 min, absorbance at 670 nm was measured with a microplate reader (Bio-Tek Instrument INC., Rockville, MD, USA). Aliquots (500 μl) of mouse plasma were tested for H_2_S using the same procedure. All standards and samples were assayed in duplicate. The calibration curve of absorbance versus H_2_S concentration was obtained by using NaHS solution of varying concentrations (10 to 160 μm).

### Quantitation of atherosclerotic plaque

Quantitation of atherosclerotic plaque was carried out as previously reported [5]. In brief, atherosclerotic lesions in the aortic root were examined at three location levels, and nine serial sections were prepared from each location. Sections from three locations were selected and stained with oil red O and counterstained with Mayer’s hematoxylin. The images were then produced using an inverted microscope (Olympus IX-83, Tokyo, Japan). Histological staining in all images was quantified using ImageJ, and the mean staining within the aortic root of each mouse was obtained.

### Detection of S-sulfhydration by modified biotin switch assay

The assay was carried out with modifications according to Mustafa *et al*. [8]. Control and H_2_S-treated ApoE KO mice aortas were homogenised in HENS buffer [250 mM HEPES-NaOH (pH 7.7), 1 mM EDTA, and 0.1 mM neocuproine] supplemented with 100 μM deferoxamine and centrifuged at 13,000 *g* for 30 min at 4°C. Aorta homogenates were added to blocking buffer [HENS buffer adjusted to 2.5% sodium dodecyl sulfate (SDS) and 20 mM methyl methanethiosulfonate (MMTS)] at 50°C for 30 min with rotation. MMTS was removed by acetone, and the proteins were precipitated at –20°C for 30 min. After acetone removal, the proteins were resuspended in HENS buffer with 1% SDS and then incubated for 3 h at room temperature with biotin-HPDP in dimethyl sulfoxide without ascorbic acid, centrifuged, and resuspended in HENS buffer. The biotinylated and *S*-sulfhydrated proteins were pulled down by streptavidin beads. To detect biotinylated proteins, samples were separated on 12% SDS-PAGE gels, transferred to a polyvinylidene difluoride (PVDF) membrane, blocked with 5% non-fat milk, and incubated with streptavidin-peroxidase diluted 1:2000 for 1 h. The biotinylated proteins were also subjected to western blotting analysis using anti-glutathione peroxidase 1 (anti-GPx1) antibody (Abcam, Cambridge, UK).

### In-gel tryptic digestion

The biotinylated proteins were separated on a 12% SDS polyacrylamide gel (SDS-PAGE) and stained with coomassie blue. The gel lanes were cut into three slices. Each slice was further cut into 1x1 to 2x2-mm pieces, which were then placed into a 1.5 mL tube. The gel pieces were destained two to three times with destaining solution (50% acetonitrile, 25 mM ammonium bicarbonate). After in-gel reduction (10 mM dithiothreitol, 45 min, 56°C) and alkylation (11 mM iodoacetamide, 45 min in dark), gel pieces were shrunk in acetonitrite and dried down. Each gel slice sample was rehydrated by 0.3 μg trypsin in 25 mM ammonium bicarbonate and incubated overnight at 37°C. After that, the reaction was stopped with 1% trifluoroacetic acid. Peptides were extracted from gel pieces with incubation in extraction buffer (50% acetonitrile, 1% trifluoroacetic acid). The supernatant from all the fractions of the same sample were pooled and dried using a Speedvac.

### Mass spectrometry analysis and protein identification

The peptides were dissolved in a solution of 1% formic acid in water and injected onto an LC/MS system consisting of a Dionex Ultimate 3000 nano-LC system (Thermo Fisher Scientific Inc., Germany) associated with fraction collector, Proteineer fcII (Bruker Daltonics, Bremen, Germany) to fractionate and spot fractions onto MALDI target plates for protein identification for matrix-assisted laser desorption/ionisation. Mass spectrometry analyses were carried out on an UltrafleXtreme MALDI TOF/TOF analyser (Bruker Daltonics; Bremen, Germany). The database search was done using the Mascot search engine (Matrix Science Mascot 2.4.1) on the SwissProt databases (545536 sequences). The following parameters were used: up to 1 miss cleavages; MS tolerance 50 ppm; MSMS tolerance 0.5 Da; full tryptic peptides; partial modifications carbamidomethylation (C), oxidation (M); MudPIT scoring; significance threshold *P* < 0.05. Protein identifications were validated only if they satisfied three requirements: (i) their score was significant (*P* < 0.05) with cut-off criteria; (ii) they were identified with three peptides with score >70; and (iii) they were identified in at least two out of the three runs.

### Gene ontology (GO) and enrichment analysis in the protein data set

To determine GO annotations for selected proteins, we used the Database for Annotation, Visualization and Integrated Discovery (DAVID) (https://david.ncifcrf.gov/; version 6.8) to access a relational database of gene functional annotations [14,15]. For the enrichment analysis, Protein ANalysis THrough Evolutionary Relationships (PANTHER) classification (http://geneontology.org; version 11.1) was performed to investigate the enrichment of molecular function, biological process and protein class associated with each identified protein [16]. Enriched pathways were analysed by KEGG pathway analysis using WEB-based Gene SeT AnaLysis Toolkit (WEBGESTALT) (http://www.webgestalt.org/option.php) [17]. Enriched KEGG pathways and GO terms were determined based on their corresponding *P*-values for over-representation against the entire mouse genome. *P* values were calculated using a hypergeometric test and corrected for multiple testing with a Benjamini-Hochberg (GO terms) or Bonferroni (KEGG pathway) correction. A cut-off of 0.05 was applied.

### Western blotting

Briefly, protein samples were separated by 12% SDS-PAGE and then transferred into polyvinylidene difluoride membranes. The membranes were blocked with 5% non-fat milk and then probed overnight at 4°C with anti-GPx1 (1:1000; Abcam). This step was followed by exposure to secondary antibody for 1 h with a 1:2000 dilution of goat anti-rabbit secondary antibody (Cell Signaling Technology, USA). After washing, the membrane was developed with an ECL kit (Amersham Pharmacia Biotechnology, UK) and exposed on a ChemiDoc imaging system (Bio-Rad Laboratories, Hercules, CA). Intensity of bands was quantified using the public domain software, ImageJ software with linear signal tool. Each GPx1 band of each mouse was first normalized with its actin intensity and the SH-GPx1 was then calculated (SH-GPx1/Input-GPx1).

### Immunofluorescence staining

Immunofluorescence staining of aortic root sections was performed using rabbit anti-mouse GPx1 antibody to observe the intensity of GPx1 in the aortic roots. Briefly, air-dried cryostat sections (6 μm thickness) of aortic root were fixed with the pre-cooled acetone for 10 min and rinsed with 1× phosphate buffered saline (PBS) three times. Non-specific staining was blocked by incubation with 5% goat serum in 1× PBS for 30 min followed by incubation of anti-GPx1 antibody overnight at 4°C in a 1:200 dilution. After rinsing with 1× PBS, sections were incubated with Alexa 488 anti-rabbit secondary antibody (Invitrogen Molecular Probes, UK) for 1 h while nuclei were stained with DAPI in the mounting agent (Invitrogen Molecular Probes). Microscopic examination was performed on an Olympus IX-83 inverted fluorescence microscope (Olympus, Tokyo, Japan). Images were acquired by a monochrome CCD camera using CellSens software (Olympus, Tokyo, Japan).

### Glutathione activities determination

A commercially available glutathione peroxidase (GPx) assay kit (Cayman Chemical Company) was used to measure plasma GPx activity according to the manufacturer’s instructions. Glutathione (GSH) and total GSH were measured using GSH colorimetric assay (BioVision Incorporated, CA, USA). Oxidised glutathione/glutathione disulfide (GSSG) was calculated by subtraction of GSH from total GSH.

### Lipid peroxidation determination

Malondialdehyde (MDA) is a product of lipid peroxidation. Therefore, lipid peroxidation was determined by measuring MDA concentration in mice plasma using a thiobarbituric acid reactive substances (TBARS) assay kit (Cayman Chemical Company).

### Statistical analysis

All data are presented as mean ± SEM. The non-parametric Mann Whitney test was used to compare variables between two groups. Statistical significance was determined at a level of *P* < 0.05. Statistical analysis was performed with GraphPad Prism 5.0 software.

## Results

### H_2_S reduced body weight gain and aortic plaque area in ApoE KO mice

**Fig 1:**
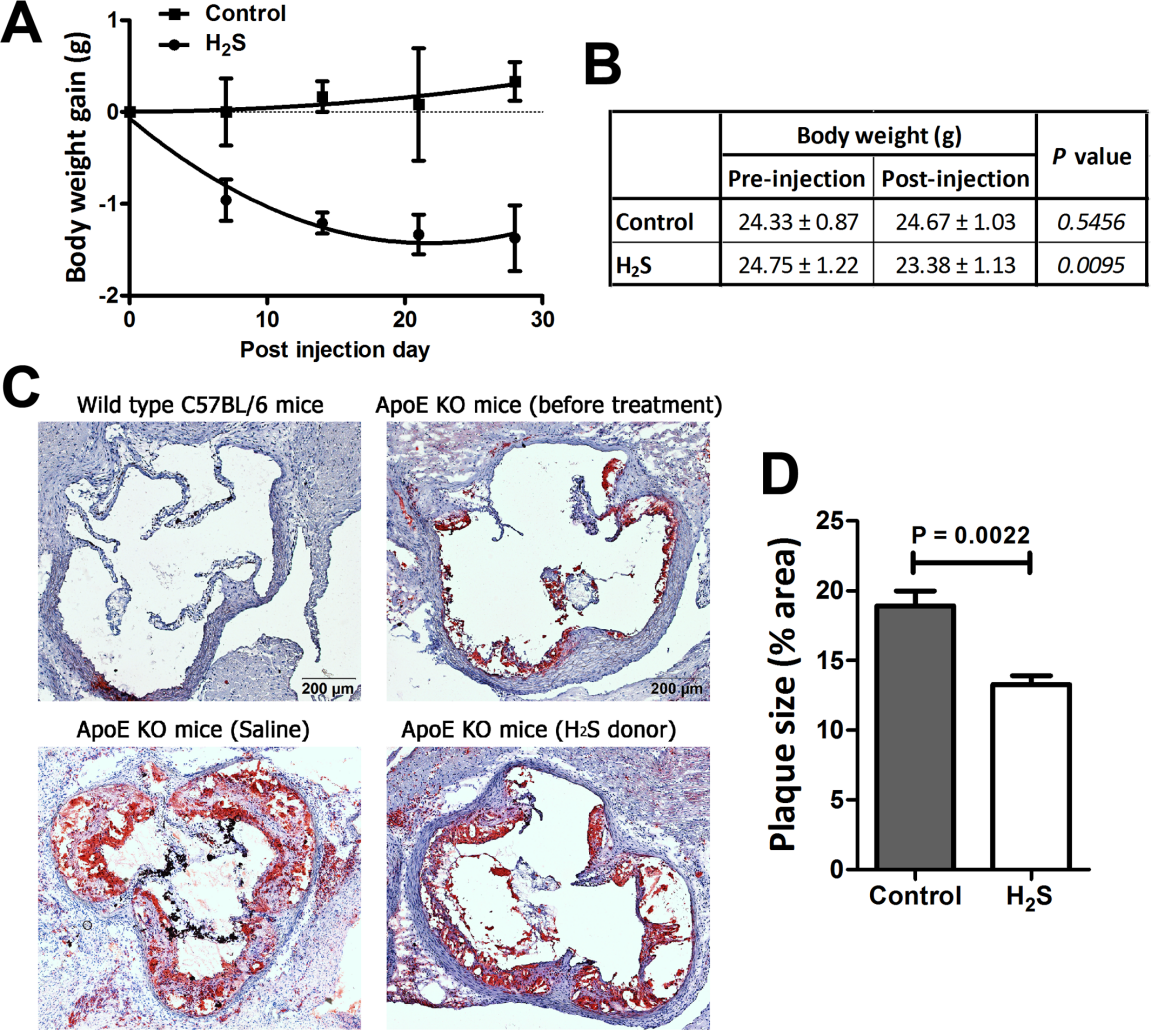
H_2_S administration in ApoE KO mice can reduce body weight gain and atherosclerotic plaque area. ApoE KO mice were first fed an atherogenic diet for 4 weeks before H_2_S treatment and then randomly divided into two groups: (i) ApoE KO + saline (0.1 mL per day, i.p.) for 4 weeks and (ii) ApoE KO + H_2_S donor (133 μmol per kg per day, i.p.) for 4 weeks. (A) During the treatment, the body weight of mice was measured weekly and the weight gain per week was calculated. Body weight was lost in mice treated with H_2_S in the first week, whereas the control group had no significant change in weight. Data represent mean ± SEM (*n* = 6–12). (B) The body weight (± SD) was compared before treatment and after 4-weeks of treated in both groups of mice (*n* = 6–12). H_2_S-treated ApoE KO mice were shown to have significant body weight loss after the H_2_S treatment. (C) After treatment, the aortic root was sectioned and stained with oil red O for lipids. H_2_S-treated ApoE KO mice showed reduced lesion area (appearing as red colour in the aortic root) as compared to the saline control group. It was also compared to the ApoE KO mice before H_2_S treatment and the WT C57BL/6J mice, which showed few lesions and no lesions in the aortic root, respectively. (D) The plaque size was quantified as percentage area of red staining in the aortic root. Values are means ± SEM, *n* = 6. The groups were significantly different from each other according to the Mann Whitney test, *P* < 0.05.

All ApoE KO mice were first fed with an atherogenic diet for 4 weeks. Their body weight gain decreased in the first week but subsequently returned to normal and remained stable. The mice were then divided into the vehicle-control group and H_2_S-treated group. The body weight gain in the H_2_S group was significantly reduced after H_2_S treatment, whereas the control group body weight remained similar over the whole period of treatment (Fig 1A). The H_2_S-treated mice had about 5% body weight loss after 4 weeks of H_2_S treatment (Fig 1B). To demonstrate whether GYY4137 treatment could account for food consumption and thus body weight gain, food uptake by both group mice was measured. It found that GYY4137 treatment did not affect the food consumption (S2 Table). In addition, after the H_2_S treatment, the aortic roots were sectioned and stained with lipids using oil red O, which stains atherosclerotic plaques a red colour. There was no red staining in WT mice, and little staining seen in the atherogenic diet-fed ApoE KO mice before treatment (Fig 1C). After treatment, comparing the size of plaques in the two groups, the control group of mice had a larger plaque size in the aortic root (Fig 1D) and the oil red O staining was widely observed in the histological result (Fig 1C). In contrast, the plaque size of H_2_S-treated mice was significantly diminished by 30% (Fig 1C and 1D), indicating the retardation of the atherosclerotic response in the mice by H_2_S treatment.

### Identification and characterisation of S-sulfhydrated proteins in aorta

**Fig 2:**
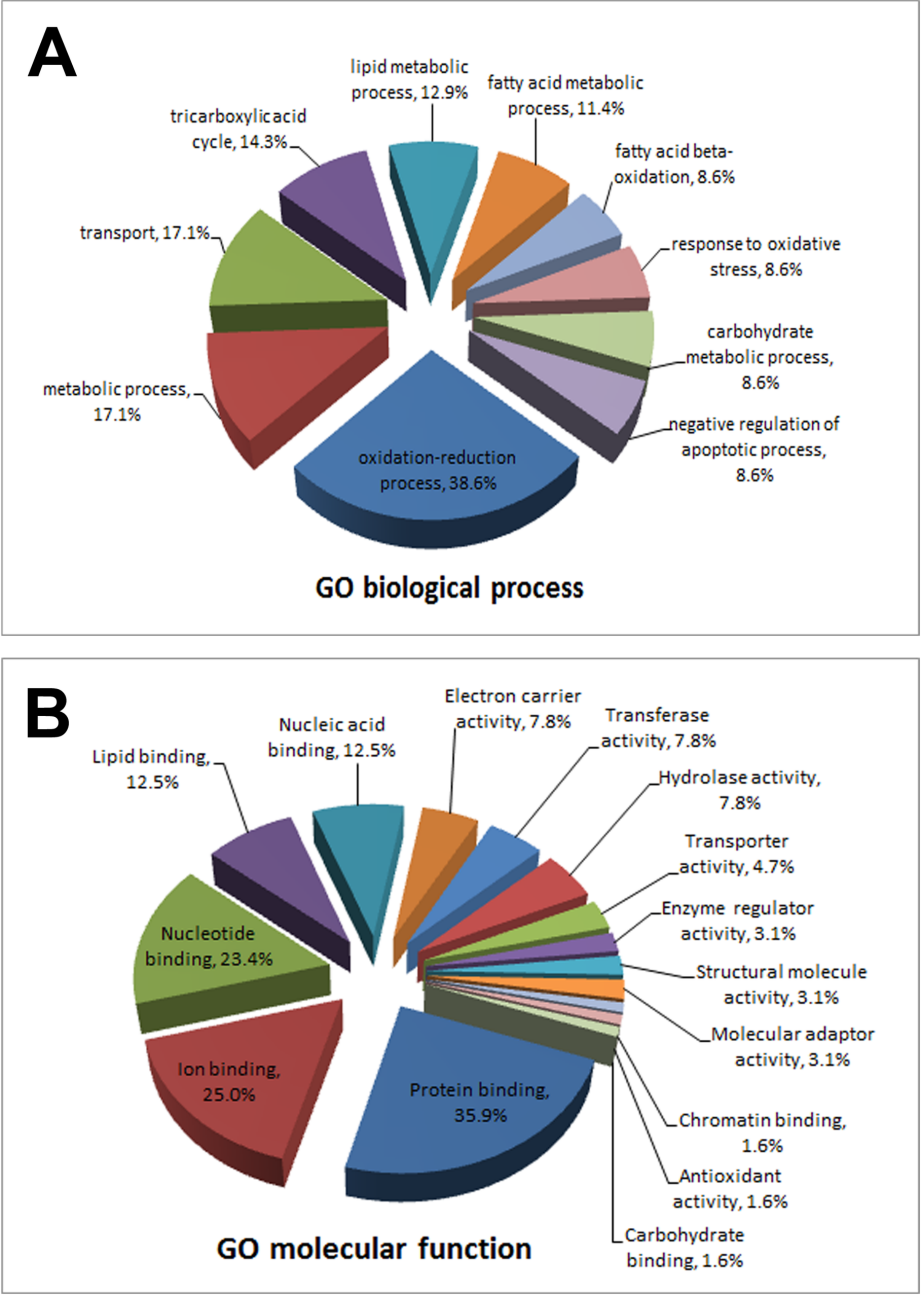
Gene ontology (GO) analysis of *S*-sulfhydrated aortic proteins. The proteins were analysed in terms of (A) GO biological process and (B) GO molecular function of proteins.

The modified approach for the biotin switch method for detecting endogenous and exogenous H_2_S revealed a total of 70 significant proteins (*P* values < 0.05), corresponding to different *S*-sulfhydrated proteins identified using tandem mass spectrometry (Table 1). For the 70 identified and *S*-sulfhydrated proteins, GO analysis showed that 38.6% of the identified proteins were involved in oxidation-reduction processes. Other biological processes involved transport (17.1%), metabolic process (17.1%), tricarboxylic acid cycle (TCA; 14.3%), lipid metabolic process (12.9%), fatty acid metabolic process (11.4%), response to oxidative stress (8.6%) and negative regulation of apoptotic process (8.6%) (Fig 2A). In regard to molecular function, the identified proteins are responsible for protein binding (35.9%), ion binding (25.0%), nucleotide binding (23.4%), lipid binding (12.5%), enzyme regulator activity (3.1%) and also antioxidant activity (1.6%) (Fig 2B).

**Table 1:** Identified *S*-sulfhydrated proteins from aorta of ApoE KO mice treated with H_2_S.

No.	Accession	ID	Protein	MW [kDa]	pI	Score	No. of Peptides	Coverage [%]
1	P60710	ACTB	Actin, cytoplasmic 1	41.7	5.3	871.6	13	43.2
2	P63268	ACTH	Actin, gamma-enteric smooth muscle	41.8	5.3	702.6	13	43.9
3	P10605	CATB	Cathepsin B	37.3	5.6	520.1	8	20.9
4	Q8BWT1	THIM	3-ketoacyl-CoA thiolase, mitochondrial	41.8	8.3	508.7	10	27
5	Q9DBJ1	PGAM1	Phosphoglycerate mutase 1	28.8	6.7	447.4	7	32.3
6	Q9DCW4	ETFB	Electron transfer flavoprotein subunit beta	27.6	8.2	442.5	6	34.5
7	Q8BFZ3	ACTBL	Beta-actin-like protein 2	42.0	5.3	420.7	7	20.5
8	P17742	PPIA	Peptidyl-prolyl *cis*-*trans* isomerase A	18.0	7.7	366.5	7	43.9
9	P07356	ANXA2	Annexin A2	38.7	7.5	348.5	5	22.4
10	P16015	CAH3	Carbonic anhydrase 3	29.3	6.9	314.6	6	30.8
11	Q05816	FABP5	Fatty acid-binding protein, epidermal	15.1	6.1	310	5	52.6
12	P05064	ALDOA	Fructose-bisphosphate aldolase A	39.3	8.3	309.1	6	20.3
13	P62962	PROF1	Profilin-1	14.9	8.5	304.3	5	40.0
14	P35486	ODPA	Pyruvate dehydrogenase E1 component subunit alpha, somatic form, mitochondrial	43.2	8.5	295.8	6	16.9
15	O88569	ROA2	Heterogeneous nuclear ribonucleoproteins A2/B1	37.4	9.0	294.4	4	19.3
16	Q9CZU6	CISY	Citrate synthase, mitochondrial	51.7	8.7	294.1	6	16.8
17	P04117	FABP4	Fatty acid-binding protein, adipocyte	14.6	8.5	284.8	4	26.5
18	P01942	HBA	Hemoglobin subunit alpha	15.1	8.0	277.8	4	34.5
19	P97429	ANXA4	Annexin A4	35.9	5.4	271.9	6	22.9
20	P19783	COX41	Cytochrome *c* oxidase subunit 4 isoform 1, mitochondrial	19.5	9.3	271.2	6	28.4
21	Q8K1Z0	COQ9	Ubiquinone biosynthesis protein COQ9, mitochondrial	35.1	5.6	270.5	3	18.2
22	Q9WVA4	TAGL2	Transgelin-2	22.4	8.4	257.2	4	28.1
23	Q01768	NDKB	Nucleoside diphosphate kinase B	17.4	7.0	257.0	6	42.1
24	P54071	IDHP	Isocitrate dehydrogenase [NADP], mitochondrial	50.9	8.9	253.9	6	15.3
25	Q9D6R2	IDH3A	Isocitrate dehydrogenase [NAD] subunit alpha, mitochondrial	39.6	6.3	248.2	6	17.2
26	P70404	IDHG1	Isocitrate dehydrogenase [NAD] subunit gamma 1, mitochondrial	42.8	9.2	245.1	4	14.0
27	P51174	ACADL	Long-chain specific acyl-CoA dehydrogenase, mitochondrial	47.9	8.5	244.3	6	17.0
28	P20152	VIME	Vimentin	53.7	5.1	234.3	5	16.1
29	Q9D051	ODPB	Pyruvate dehydrogenase E1 component subunit beta, mitochondrial	38.9	6.4	228.5	5	25.6
30	Q8BH95	ECHM	Enoyl-CoA hydratase, mitochondrial	31.5	8.8	219.3	3	15.2
31	P14152	MDHC	Malate dehydrogenase, cytoplasmic	36.5	6.2	214.2	5	18.9
32	Q7TMF3	NDUAC	NADH dehydrogenase [ubiquinone] 1 alpha subcomplex subunit 12	17.1	9.4	213.9	5	45.5
33	P48036	ANXA5	Annexin A5	35.7	4.8	212.5	5	22.3
34	P10649	GSTM1	Glutathione *S*-transferase Mu 1	26.0	7.7	208.6	5	25.2
35	Q99LY9	NDUS5	NADH dehydrogenase [ubiquinone] iron-sulfur protein 5	12.6	9.1	207.3	4	46.2
36	P08249	MDHM	Malate dehydrogenase, mitochondrial	35.6	8.9	205.3	4	16.0
37	O08756	HCD2	3-hydroxyacyl-CoA dehydrogenase type-2	27.4	8.5	201.2	4	19.9
38	Q61425	HCDH	Hydroxyacyl-coenzyme A dehydrogenase, mitochondrial	34.4	8.8	196.2	4	10.8
39	Q9CZ13	QCR1	Cytochrome *b-c*1 complex subunit 1, mitochondrial	52.8	5.8	195.7	4	14.6
40	P10107	ANXA1	Annexin A1	38.7	7.0	193.0	4	13.6
41	P09671	SODM	Superoxide dismutase [Mn], mitochondrial	24.6	8.8	176.3	3	19.8
42	P02088	HBB1	Hemoglobin subunit beta-1	15.8	7.1	156.4	3	21.8
43	P99024	TBB5	Tubulin beta-5 chain	49.6	4.8	156.1	5	11.9
44	Q9CR61	NDUB7	NADH dehydrogenase [ubiquinone] 1 beta subcomplex subunit 7	16.3	8.4	153.1	3	38.0
45	O08807	PRDX4	Peroxiredoxin-4	31.0	6.7	149.7	3	11.7
46	Q9Z2I9	SUCB1	Succinyl-CoA ligase [ADP-forming] subunit beta, mitochondrial	50.1	6.6	148.0	4	9.7
47	Q9D0M3	CY1	Cytochrome *c*1, heme protein, mitochondrial	35.3	9.2	142.9	3	15.4
48	P13707	GPDA	Glycerol-3-phosphate dehydrogenase [NAD(+)], cytoplasmic	37.5	6.8	138	4	12.0
49	O35855	BCAT2	Branched-chain-amino-acid aminotransferase, mitochondrial	44.1	8.6	137.8	4	14.0
50	P14131	RS16	40S ribosomal protein S16	16.4	10.2	133.2	4	24.7
51	Q99JY0	ECHB	Trifunctional enzyme subunit beta, mitochondrial	51.4	9.4	133.0	4	9.9
52	P67778	PHB	Prohibitin	29.8	5.6	131.5	3	16.2
53	Q9DC69	NDUA9	NADH dehydrogenase [ubiquinone] 1 alpha subcomplex subunit 9, mitochondrial	42.5	9.7	131.2	3	8.0
54	P09528	FRIH	Ferritin heavy chain	21.1	5.5	128.5	3	15.4
55	Q9CQI6	COTL1	Coactosin-like protein	15.9	5.2	128	4	23.9
56	P42125	ECI1	Enoyl-CoA delta isomerase 1, mitochondrial	32.2	9.1	127.1	3	9.3
57	Q9R0P5	DEST	Destrin	18.5	8.1	124.4	3	18.2
58	P15105	GLNA	Glutamine synthetase	42.1	6.6	123.5	3	12.3
59	Q8BFR5	EFTU	Elongation factor Tu, mitochondrial	49.5	7.2	117.7	3	10.0
60	P16110	LEG3	Galectin-3	27.5	8.5	115.5	3	12.1
61	P14206	RSSA	40S ribosomal protein SA	32.8	4.8	113.8	4	15.3
62	Q9DCJ5	NDUA8	NADH dehydrogenase [ubiquinone] 1 alpha subcomplex subunit 8	20.0	8.8	110.1	3	18.6
63	Q9JII6	AK1A1	Alcohol dehydrogenase [NADP(+)]	36.6	6.9	97.3	3	6.2
64	P97351	RS3A	40S ribosomal protein S3a	29.9	9.8	92.5	3	14.8
65	P45952	ACADM	Medium-chain specific acyl-CoA dehydrogenase, mitochondrial	46.5	8.6	91.7	3	5.9
66	P59999	ARPC4	Actin-related protein 2/3 complex subunit 4	19.7	8.5	87.4	3	22.6
67	O88844	IDHC	Isocitrate dehydrogenase [NADP] cytoplasmic	46.6	6.7	85.5	3	9.4
68	P35700	PRDX1	Peroxiredoxin-1	22.2	8.3	81.0	3	22.6
69	P61205	ARF3	ADP-ribosylation factor 3	20.6	6.8	80.5	3	14.9
70	P11352	GPx1	Glutathione peroxidase 1	22.3	6.7	71.4	3	17.4

### Enrichment analysis of S-sulfhydrated proteins in aorta

**Fig 3:**
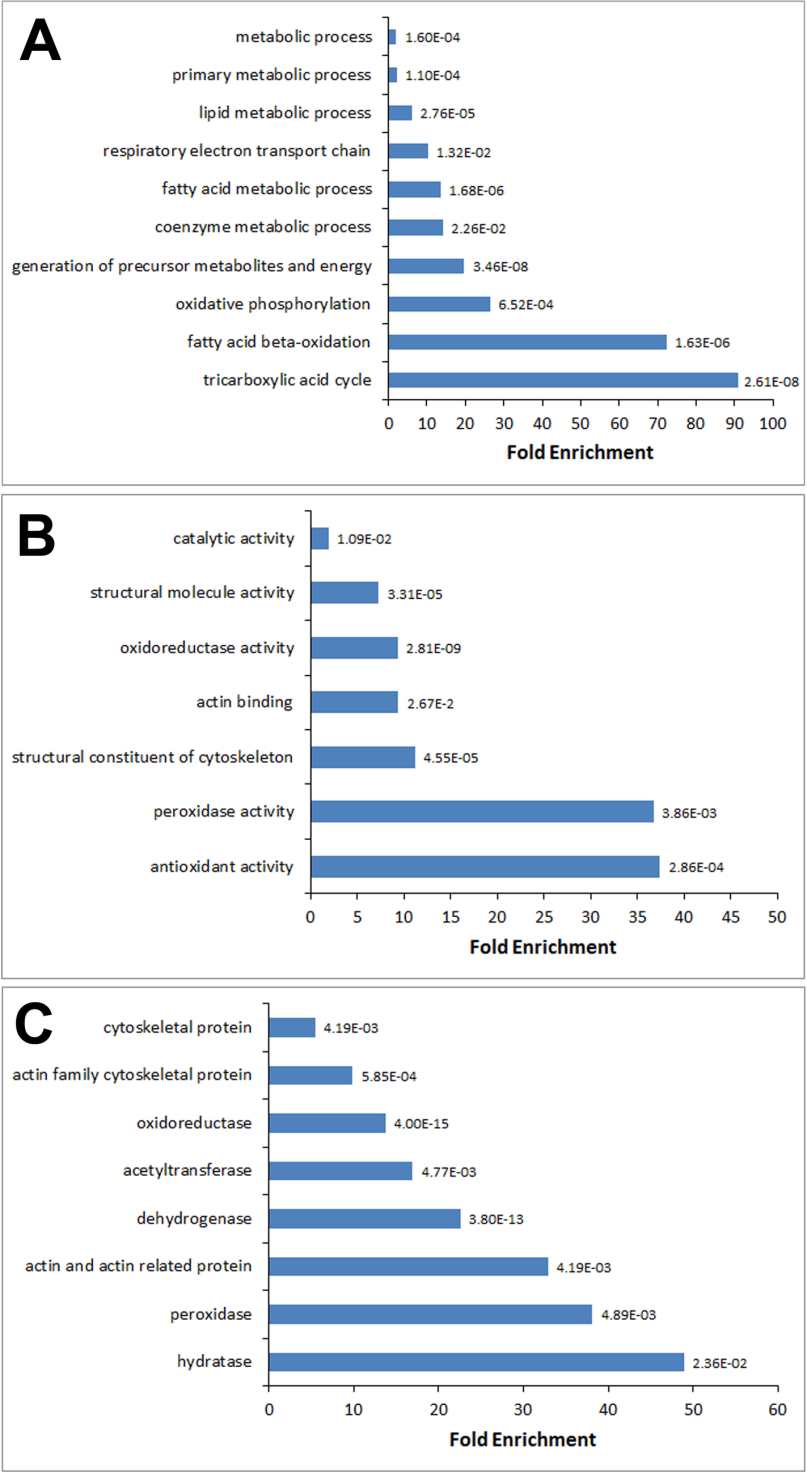
Gene ontology (GO) enrichment analysis for *S*-sulfhydrated proteins in mice aorta. Enriched GO terms for (A) biological process, (B) molecular function and (C) protein class were determined based on their corresponding *P* values for over-representation against the mice entire genome.

In the enrichment analysis, biological processes categorised as lipid metabolism (e.g., fatty acid metabolism and oxidation) or energy production and conversion (e.g., TCA cycle) were mainly enriched in the identified protein set (Fig 3A). For the molecular function, proteins involved in antioxidant activity were enriched the most against the entire mice genome, followed by peroxidase activity (Fig 3B). In terms of protein class, hydratase was augmented the most in the identified proteins (~ 50-fold). Others included peroxidase (~38-fold), actin-related proteins (~33-fold) and dehydrogenase (~23-fold) (Fig 3C).

**Fig 4:**
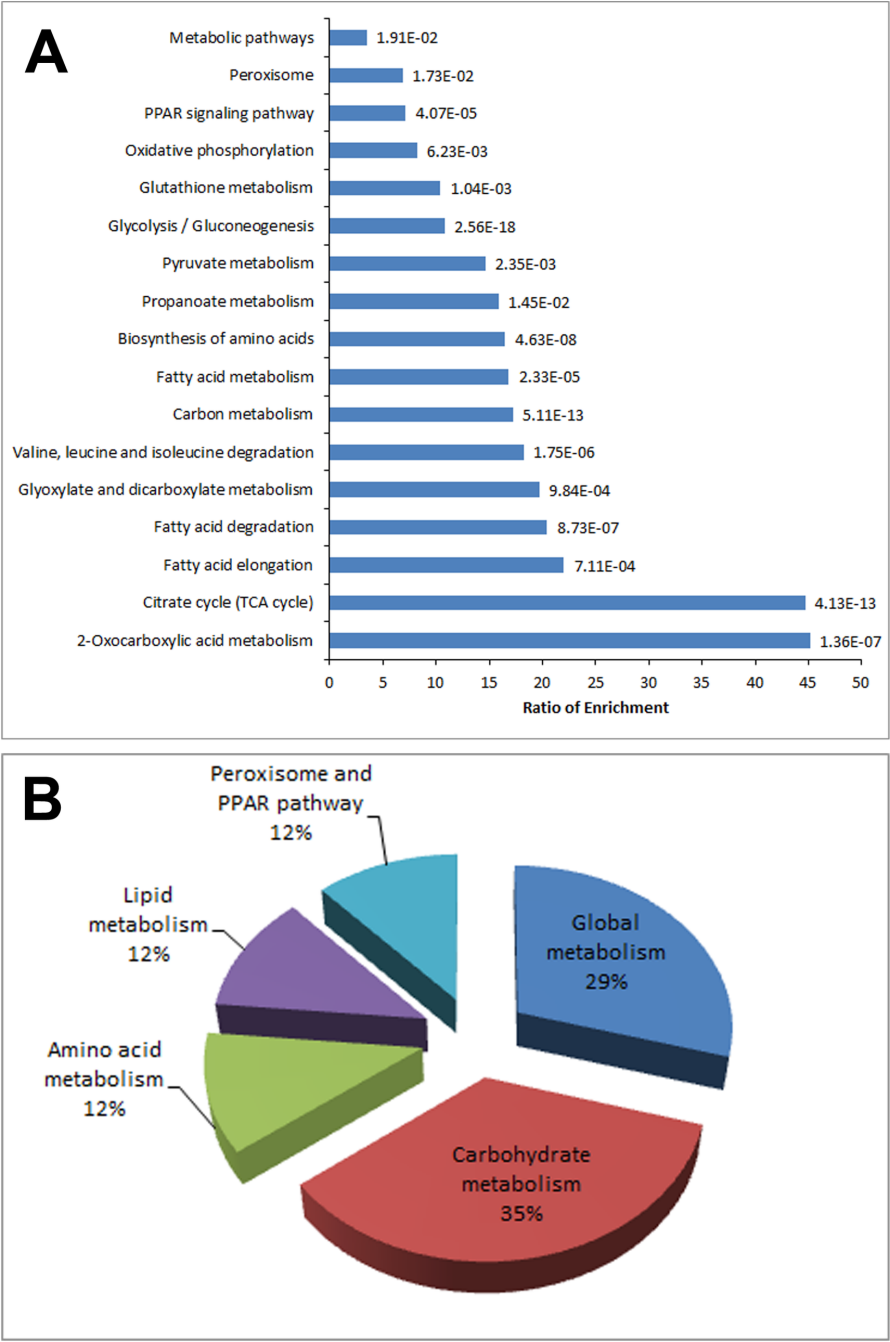
Pathway enrichment analyses for *S*-sulfhydrated proteins in mice aorta. (A) 19 KEGG pathways were found to be enriched, and the ratio of enrichment of each pathway was revealed in the identified protein set of *S*-sulfhydration with their corresponding *P* values. (B) The enriched KEGG pathways were categorised and presented as percentage that the largest group of the proteins were constituted by global metabolism. Lipid metabolism and amino acid metabolism were also major contributors, whereas a number of pathways were classified as ‘others’.

Furthermore, the enrichment of identified *S*-sulfhyrated proteins was observed in 17 molecular pathways by the pathway enrichment analysis using KEGG (WEBGESTALT) (Fig 4A). Out of 70 sulfhydrated proteins, 5 were involved in lipid metabolism pathways, including hydroxyacyl-coenzyme A dehydrogenase (HADH), acyl-CoA dehydrogenases, long chain (ACADL), enoyl CoA hydratase, short chain, 1, mitochondrial (ECHS1) acyl-CoA dehydrogenase, medium chain (ACADM) and enoyl-CoA delta isomerase 1 (ECI1). Also found to be enriched were proteins involved in valine/leucine/isoleucine (e.g., ACADM and branched-chain-amino-acid aminotransferase, mitochondrial, BCAT2) and GSH metabolism (e.g., GPx1 and glutathione *S*-transferase Mu 1, GSTM1). The peroxisome and peroxisome proliferator-activated receptors (PPARs) signalling pathway was also found to be augmented and could play a role in redox signalling and lipid homoeostasis. Enrichment of identified *S*-sulfhydrated proteins was also observed in the TCA cycle, pyruvate metabolism and glycolysis (Fig 4A). Most of the enriched pathways were metabolic pathways involved in energy production and conversion. Other pathways of lipid metabolism and amino acid metabolism were also observed to be elevated (Fig 4B), indicating that H_2_S appears to mitigate atherosclerosis through regulation of lipid metabolism and anti-oxidative response to stimulus.

### Protein S-sulfhydration of aortic proteins

**Fig 5:**
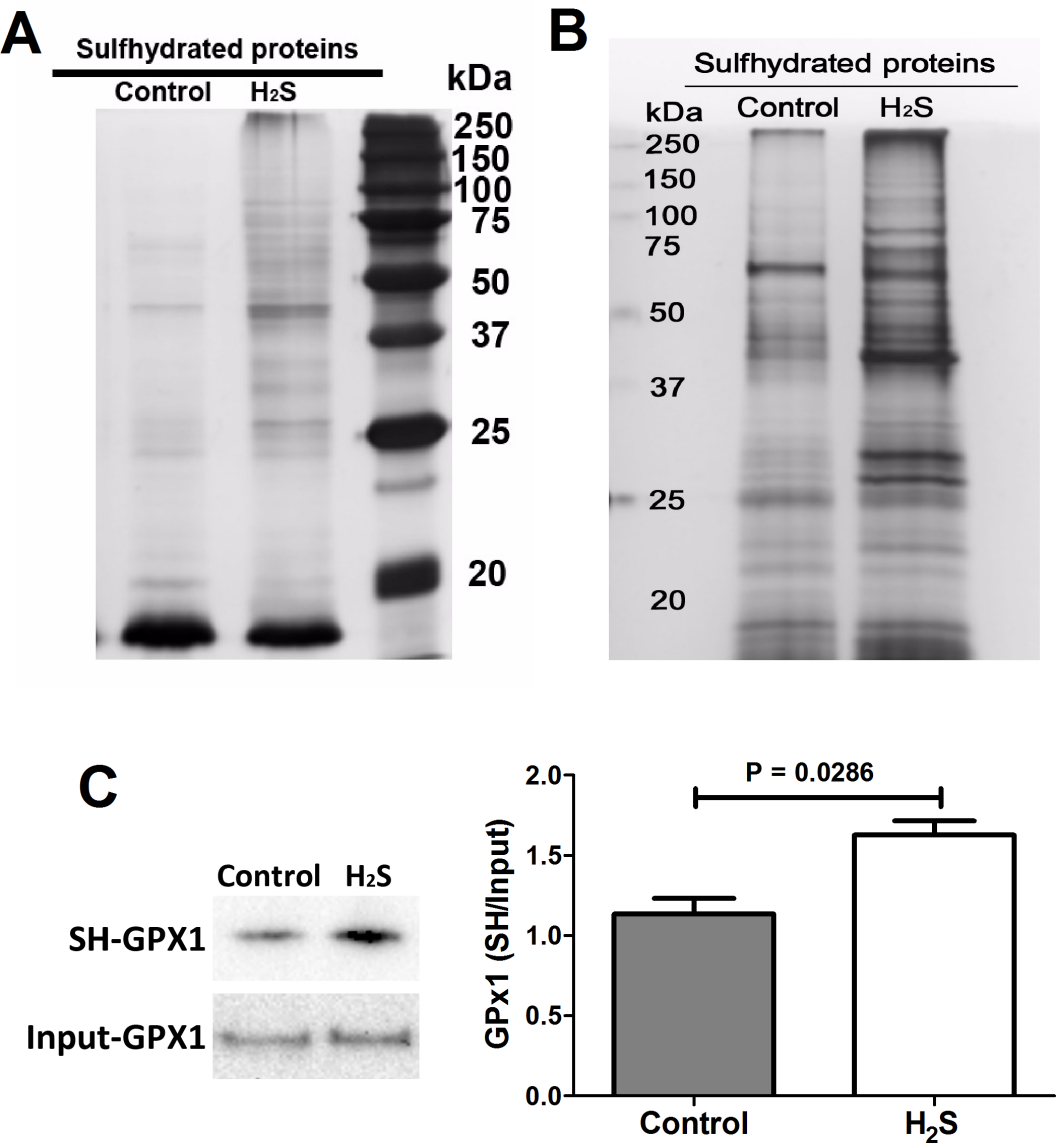
H_2_S effects on *S*-sulfhydrated proteins and GPx1 *S*-sulfhydration. (A) Gel staining of biotinylated proteins in control and H_2_S-treated ApoE KO mice. (B) Detection of *S*-sulfhydrated proteins by western blot analysis in control and H_2_S-treated ApoE KO mice. There was an increase of *S*-sulfhydration upon H_2_S treatment. (C) Representative blot image of immunoprecipitated GPx1 from control and H_2_S-treated mice aorta was extracted from the blot of first mice pair (control and H_2_S-treated mice). H_2_S sulfhydrated GPx1 overexpressed in the aorta lysate of H_2_S-treated mice and the band intensities were quantified using ImageJ. SH–GPx1, *S*-sulfhydrated GPx1; input-GPx1, total GPx1. Comparison between groups by Mann Whitney test, *P* < 0.05; *n* = 4 for each group.

H_2_S signalling through *S*-sulfhydration involves H_2_S altering functions of proteins by modification on cysteine residues in proteins through the formation of a persulfide (–SSH) bond [18]. A modified biotin switch method, a way to detect *S*-sulfhydrated proteins, was used to determine the pattern of *S*-sulfhydration of aortic proteins in control and H_2_S-treated mice. The results indicated that H_2_S treatment induced an increase in *S*-sulfhydration of aorta proteins as compared to the controls (Fig 5A). The *S*-sulfhydrated proteins appeared as bands on western blotting from low-molecular-weight proteins to high-molecular-weight proteins (Fig 5B), including the band corresponding to GPx1, which is involved in cellular response to stress during atherosclerosis. Western blot analysis of aorta lysate revealed a band corresponding to GPx1 at 22 kDa as a putative target for *S*-sulfhydration (Fig 5C). This was confirmed by immunoprecipitation of the aorta proteins with anti-GPx1 antibody on both control and H_2_S-treated aortas. We observed that endogenous GPx1 *S*-sulfhydration increased by 1.4-fold upon H_2_S treatment (Fig 5C), suggesting that H_2_S can *S*-sulfhydrate GPx1 proteins and that *S*-sulfhydration was enhanced after H_2_S treatment.

### S-sulfhydration reduced lipid peroxidation and enhanced GSH synthesis

**Fig 6:**
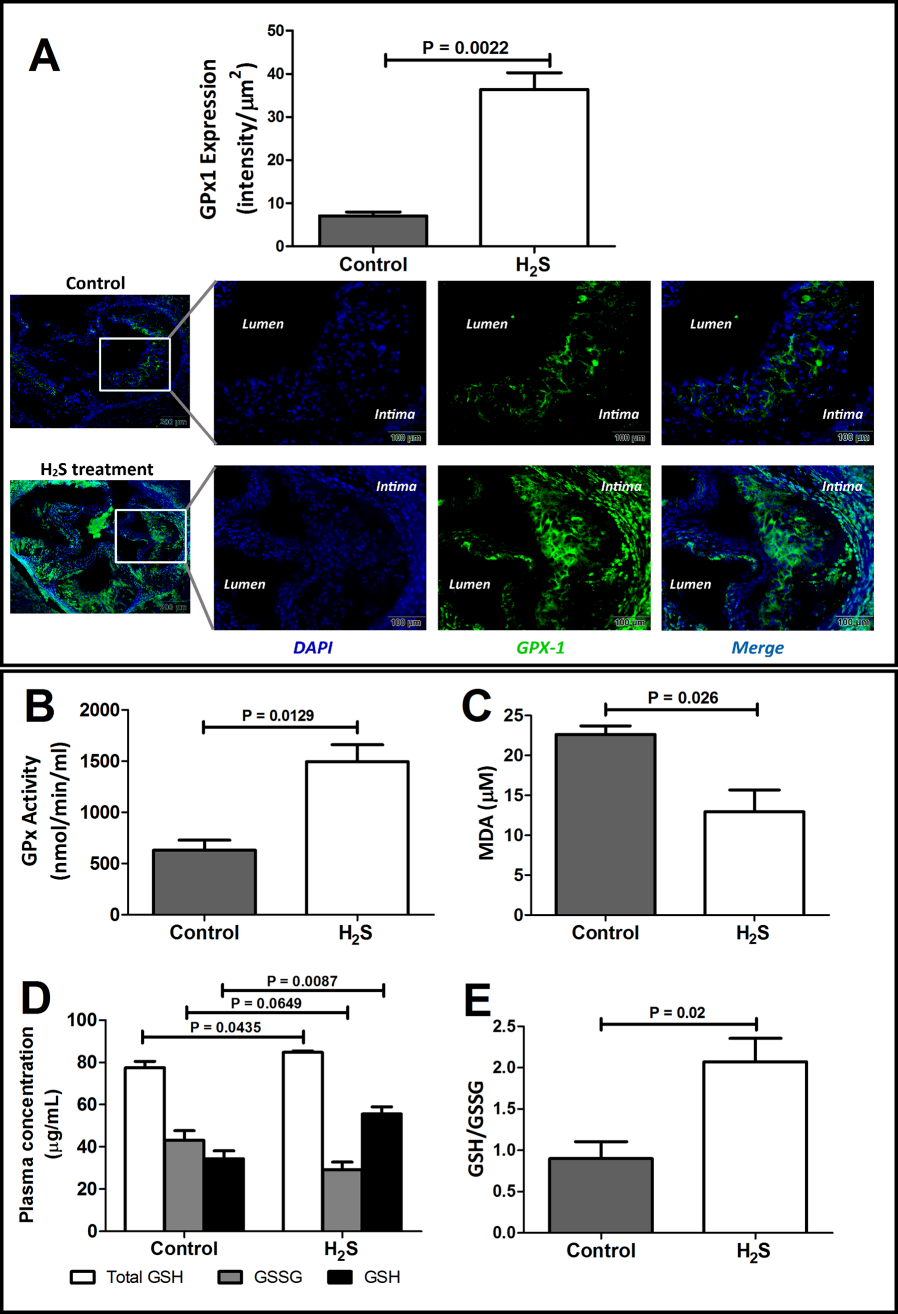
*S*-sulfhydration enhances anti-oxidative stress responses. (A) Immunofluorescence staining of GPx1 in aortic root of ApoE KO mice with or without H_2_S treatment. It showed overexpression of GPx1 in aortic root lesions and intima of aortic root sections after 4 weeks of H_2_S administration. GPx1 appeared as green fluorescence, whereas cell nuclei are represented by blue fluorescence of DAPI. The images are shown at 10× magnification. The GPx1 signals in aortic roots were quantified by ImageJ software. (B) H_2_S treatment significantly increased plasma GPx activity (oxidation of NADPH to NADP^+^), suggesting inhibition of oxidative damage. (C) Lipid peroxidation was determined by measurement of malondialdehyde (MDA) in plasma. MDA level was reduced in H_2_S-treated mice. (D) Plasma glutathione (GSH) assay significantly showed increase of total GSH and GSH but decreased GSSG, indicating the cells were protected from oxidative damage. (E) Higher ratio of GSH/GSSG in H_2_S treatment group also indicated antioxidant effects of H_2_S. GPx, glutathione peroxidase; GSH, glutathione; GSSG, oxidised glutathione. Values are means ± SEM, *n* = 6. The groups are significantly different from each other by Mann Whitney test, *P* < 0.05.

To confirm the effect of *S*-sulfhydration on aortic tissue, we examined the expression and activity of GPx1. GPx1 was abundantly expressed as green fluorescence signal in the aortic root of H_2_S-treated mice, whereas only minimal green fluorescence was observed in the control aortic sections (Fig 6A). In addition, the plasma GPx1 activity also revealed a higher level in H_2_S-treated mice, that was about 2.4-fold higher than the activity observed in control mice (Fig 6B). These results show that *S*-sulfhydration can enhance the level of GPx1 and thus its activity. It is known that GPx is an enzyme family with peroxidase activity whose main biological role is to protect the organism from oxidative damage. It converts toxic lipid peroxides into inert alcohols and reduces free hydrogen peroxide to water by using reduced GSH. Thus, we continued to examine the effect of H_2_S on MDA and GSH contents to determine lipid peroxides production and GSH synthesis. As shown in Fig 6C, the level of MDA, the by-product of lipid peroxidation, was increased in the mice without H_2_S treatment but was significantly reduced by 43% after H_2_S administration (Fig 6C), suggesting that H_2_S facilitates clearance of reactive oxygen species (ROS) such as free radicals and thus protects the cells from damage. In parallel, to further investigate the level of GSH in relation to H_2_S supplementation, plasma concentration of total GSH, reduced GSH (GSH) and oxidised GSH (GSSG) were measured in both groups of mice (Fig 6D). The total GSH did not differ much in either group of mice, although H_2_S-treated mice had slightly higher total GSH than the control mice. Control mice had a higher GSSG level and lower GSH level, indicating accumulation of GSSG and exposure of oxidative stress in cells. However, this relationship was reversed by H_2_S treatment, as revealed by a decreased level of GSSG and an elevation in GSH level by about two times in H_2_S-treated mice in comparison to its level in control mice (Fig 6D). Consistently, results showed that the GSH/GSSG ratio was increased by 2.3-fold in H_2_S-treated mice, as compared to that in the control group (Fig 6E), demonstrating protection of cells from oxidative damage upon H_2_S treatment.

## Discussion

H_2_S administration with GYY4137 ameliorated atherosclerosis in the present study. GYY4137 is a slow-releasing donor of H_2_S. It releases H_2_S slowly both in *vitro* and *in vivo* and it just offers about 4–5 nmol per 25 min [13]. In this study, plasma H_2_S released from GYY4137 was increased at the first 30 minutes and then returned to normal after 8 hours (S1 Fig). In addition, H_2_S was accumulated in liver and kidney very quickly upon GYY4137 treatment and maintained about 2 hours then started to decline to normal after 8 hours of injection, indicating no residue of H_2_S in liver and kidney (S2 Fig). All these showed that GYY4137 is a stable H_2_S donor and it has low toxicity which could be used to study regulatory role of H_2_S in animal model with acute diseases.

H_2_S treatment was characterised by loss of body weight and reduction of plaque formation, both of which are consistent with our previous study using CSE overexpression transgenic mice as a model [5], and also agrees with other studies [4,6]. We have identified 70 proteins that are *S*-sulfhydrated to a significant degree from aortic tissue of ApoE KO mice. Functional annotation of the *S*-sulfhydration of aortic proteins reveals a main pathway of lipid metabolism in the development of atherosclerosis. Lipid metabolism is closely linked to the metabolism of carbohydrates, which may be converted to fats. The major aspect of lipid metabolism is involved with fatty acid oxidation to produce energy or the synthesis of lipids. The first step in lipid metabolism is the hydrolysis of the lipid in the cytoplasm to produce glycerol and fatty acids, which then further undergo glycolysis and β-oxidation of fatty acid, respectively. Here we show that H_2_S may regulate glycolysis and fatty acid metabolism through protein *S*-sulfhydration as revealed in our global analysis of *S*-sulfhydrated proteins in mouse aortic tissues. Pathways of glycolysis (e.g., aldolase A, fructose-bisphosphate, ALDOA and phosphoglycerate mutase 1, PGAM1) and fatty acid metabolism/oxidation (e.g., ACADL and ACADM) were augmented in the over-representation analysis.

Enhanced glycolysis and pentose phosphate pathway could result in increased antioxidant defence, as shown in the human osteosarcoma SAOS-2 cell line with expression of TAp63α [19] and in vanadium-treated diabetic rats [20] as a result of the generation of reduced GSH, a major scavenger of ROS during the process. A recent study also indicated that the CSE/H_2_S pathway plays an important role in the regulation of glucose production through *S*-sulfhydration of pyruvate carboxylase contributing to gluconeogenesis in the liver [21]. In addition, an increase in fatty acid oxidation has been observed to reduce triglyceride content and inflammatory levels, improve insulin sensitivity in adipocytes and reduce endoplasmic reticulum stress and ROS damage in macrophages [22]. Another fate of fatty acids after oxidation is the production of shortened fatty acids such as propanoate and acetate, which can then enter the circulation, where they transit directly to the liver and serve in energy production [23]. These shortened fatty acids may play an important role in health and disease, in that they help to reduce the risk of inflammatory diseases, type 2 diabetes, obesity, heart disease and other conditions [24]. Moreover, shortened fatty acids have also been shown to aid in control of body weight and insulin sensitivity [25]. Therefore, augmented propanoate metabolism in the enrichment analysis suggests a lowering of cholesterol level [26] and protection against diet-induced obesity [27].

Further, the PPAR signalling pathway was also elevated in the *S*-sulfhydrated aortic proteins we studied, and is also involved in lipid homeostasis. Fatty acid binding protein 4, adipocyte (FABP4) and fatty acid binding protein 5, epidermal (FABP5) were identified as *S*-sulfhydrated proteins in the PPAR pathway. These two proteins involved in lipid metabolism by binding to fatty acids through activation of PPARs, which comprise three subtypes: PPARα, PPARδ/β and PPARγ. These receptors are transcription factors that control the peroxisomal β-oxidation pathway of fatty acids through regulation of the acyl-CoA oxidase. Thus, PPARs play an important role in lipid metabolism. Cheng’s group demonstrated that PPARδ activated fatty acid oxidation in cultured cardiomyocytes by regulating key genes of fatty acid oxidation [28]. PPARδ was shown to act as a regulator of fat burning and was identified as a potential target in the treatment of obesity and its associated disorders [29]. In addition, PPAR was sulfhydrated after H_2_S treatment [30] and its activation could improve lipid profiles and reduce adiposity [31]. In line with this observation, recent evidence has shown that a PPARδ agonist inhibited aortic inflammation and attenuated the progression of pre-established atherosclerosis through reduction of plasma lipids, particularly very low-density and intermediate-density lipoproteins [32]. Taken together, all these studies reveal that H_2_S is highly associated with reduced obesity by regulation of glucose and lipid homoeostasis, as supported by a recent study [33] and a review [34].

In the enrichment analysis, antioxidant activity and peroxidase activity were shown to be enriched. Therefore, GPx1 can be considered as a putative target of *S*-sulfhydration in atherosclerosis. GPx1 is a selenium-dependent enzyme that is ubiquitously expressed and protects cells against oxidative damage by reducing hydrogen peroxide and a wide range of organic peroxides with reduced GSH. As an antioxidant enzyme, GPx1 has been used as a biomarker for cardiovascular risk [35,36]. It has also been proposed to be a therapeutic target for chronic obstructive pulmonary disease [37]. In atherosclerosis, increased oxidative stress is a result of the generation of ROS and increased free-radical production, which may play an important role in the pathogenesis and progression of atherosclerotic plaques. Increased lipid peroxidation also appears to be associated with oxidative stress in atherosclerosis [38] and diabetes [39]. In the present study, H_2_S *S*-sulfhydrated GPx1 and stimulated its activity in cells and in plasma. With enhanced GPx1 activity and enriched pathways such as GSH metabolism and glycolysis, GSH biosynthesis upregulation plays a critical role in the cellular defence against oxidative stress. GPx1 exerts its antioxidant effect to protect the cells from oxidative damage by reduction of lipid peroxidation and clearance of free radicals in the progression of atherosclerosis. Furthermore, enhanced GSH biosynthesis was found to be closely connected with increased fatty acid oxidation and synthesis [40] as well as reduction of body weight, as shown in our previous study [5] and another study [41].

In conclusion, the present study identified *S*-sulfhydrated proteins in several metabolic pathways. The *S*-sulfhydration level of altered proteins in aorta indicates significant protein modifications that may lead to structural or functional alterations. Protein *S*-sulfhydration in atherosclerosis targets particular enzymes participating in glycolysis, TCA cycle, and fatty acid oxidation, indicating that this post-translational modification may regulate metabolism and mitochondrial bioenergetics. Our results showed that H_2_S has such cardiovascular protective effects as prevention of body weight gain and reduction of atherosclerotic plaque formation through augmentation of lipid metabolism. H_2_S is also mediated by its antioxidant effect via GPx1 *S*-sulfhydration, which causes decreased lipid peroxidation and enhanced GSH biosynthesis. The present proteomics study provides a comprehensive analysis of H_2_S signalling via *S*-sufhydration in response to atherosclerosis.

## Supporting information

S1 Fig Plasma concentration of H_2_S in mice administered GYY4137. Blood was collected at timed intervals after GYY4137 administration and plasma H_2_S concentration was assayed. Values are means ± SEM, *n* = 3.
(TIF)

S2 Fig H_2_S concentrations in liver and kidney after GYY4137 administration. Tissues were collected at timed intervals after administration and H_2_S concentration was assayed. Values are means ± SEM, *n* = 3.
(TIF)

S1 Table A complete list of data set in this study. (PDF)

S2 Table Food uptake per mice per day. (PDF)
