# Supplementary figures and images for: Hydrogen sulfide mediates athero-protection against oxidative stress via S-sulfhydration
Source: PLoS One. 2018 Mar 8;13(3):e0194176. doi: 10.1371/journal.pone.0194176 (PMC5843340; doi:10.1371/journal.pone.0194176)

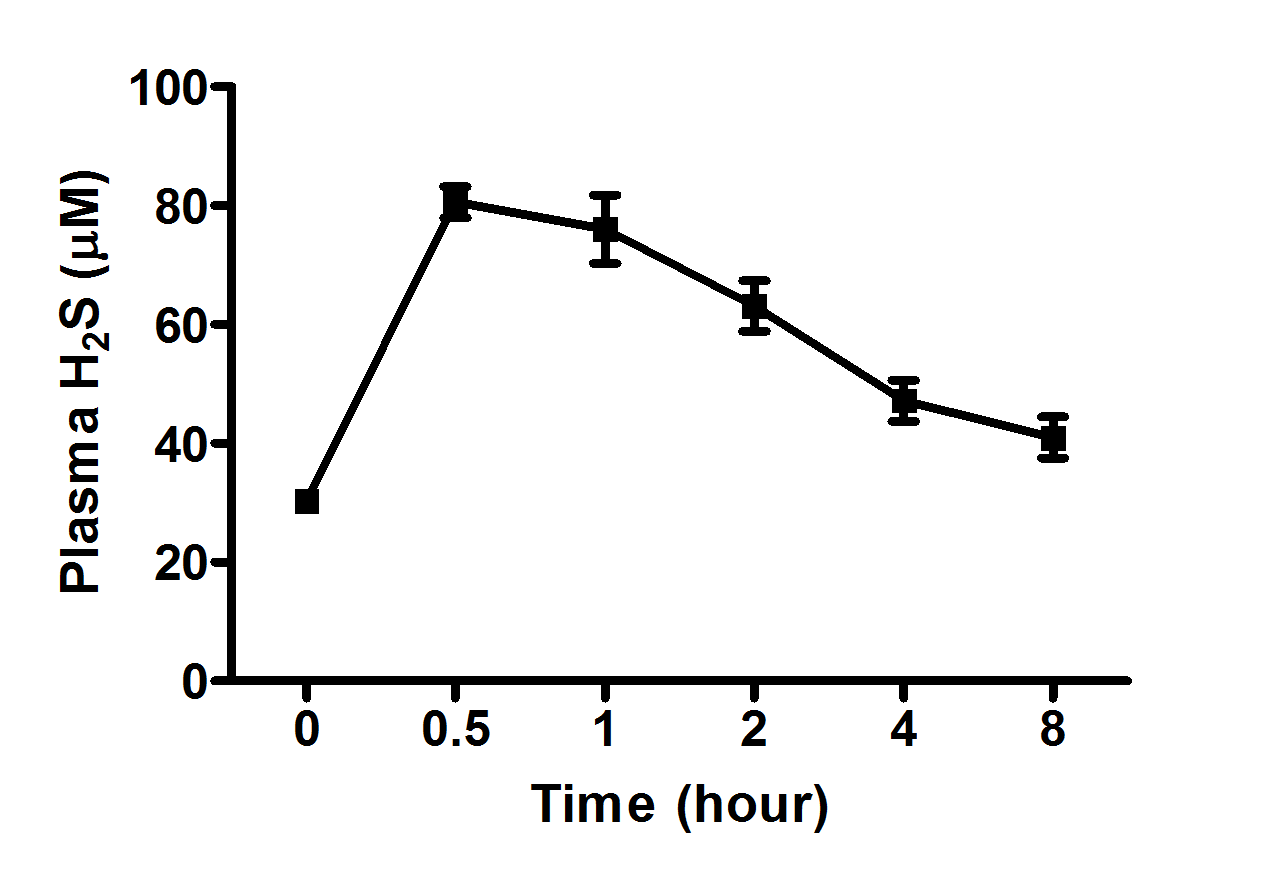

Supplement: S1 Fig — Blood was collected at timed intervals after GYY4137 administration and plasma H2S concentration was assayed. Values are means ± SEM, n = 3. (TIF) [file pone.0194176.s001.tif]

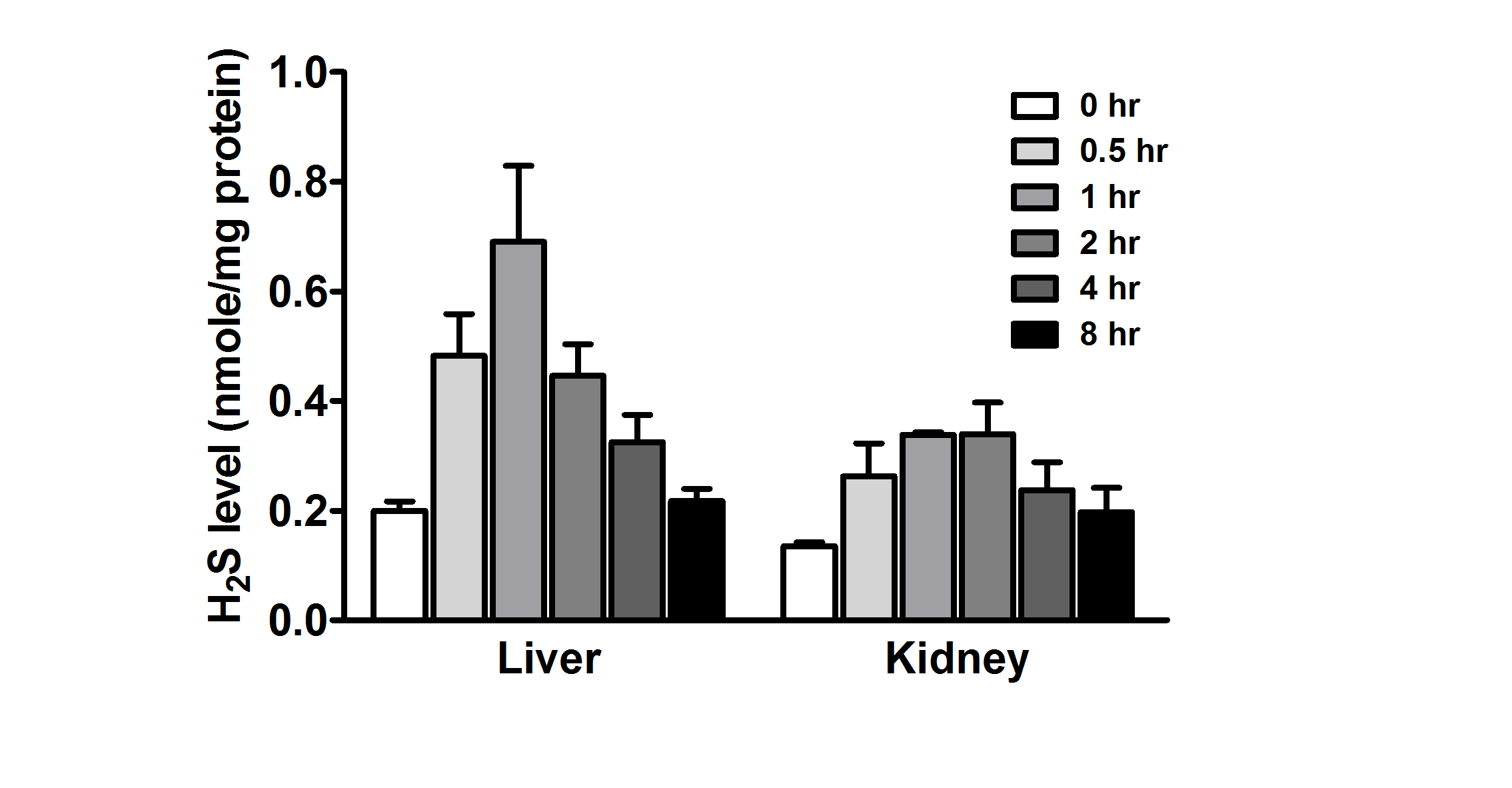

Supplement: S2 Fig — Tissues were collected at timed intervals after administration and H2S concentration was assayed. Values are means ± SEM, n = 3. (TIF) [file pone.0194176.s002.tif]
