# Supplementary material for: Hydrogen sulfide mediates athero-protection against oxidative stress via S-sulfhydration
Source: PLoS One. 2018 Mar 8;13(3):e0194176. doi: 10.1371/journal.pone.0194176 (PMC5843340; doi:10.1371/journal.pone.0194176)
Supplement: S2 Table — (PDF) [file pone.0194176.s004.pdf]

S2 Table. Food uptake per mice per day*

	**Control**	**H_2S**
Week 1	2.8	2.8
Week 2	2.7	2.7
Week 3	2.8	2.7
Week 4	2.8	2.8

* Number of mice per group is 18 and the unit is gram (g).
