# Supplementary material for: Hydrogen sulfide mediates athero-protection against oxidative stress via S-sulfhydration
Source: PLoS One. 2018 Mar 8;13(3):e0194176. doi: 10.1371/journal.pone.0194176 (PMC5843340; doi:10.1371/journal.pone.0194176)
Supplement: S1 Table — (PDF) [file pone.0194176.s003.pdf]

S1 Table. A complete list of data set in this study.

		Fig 1						Fig 5	Fig 6						
Group	Mice no.	Body weight gain					Plaque size (% area)	Sulfhydrated GPx1	GPx1 expression (staining)	GPx Activity	MDA	Total GSH	GSSG	GSH	GSH/GSSG
		Day 0	Day 7	Day 14	Day 21	Day 28									
Control	CTL-1	0.0	1.0	0.0	-2.0	0.0	18.11	0.89	5.68	636.73	21.07	79.05	56.88	22.17	0.39
	CTL-2	0.0	-1.0	0.0	-1.0	0.0	19.19	1.22	4.55	636.73	18.89	65.84	37.27	28.57	0.77
	CTL-3	0.0	0.0	0.0	1.5	0.0	17.67	1.34	5.87	891.42	21.35	84.37	52.80	31.58	0.60
	CTL-4	0.0	0.0	0.0	2.0	0.0	23.62	1.09	10.81	509.38	25.35	72.17	25.48	46.69	1.83
	CTL-5	0.0	1.0	1.0	0.0	1.0	19.13	-	8.19	254.69	25.80	79.05	44.73	34.32	0.77
	CTL-6	0.0	-1.0	0.0	0.0	1.0	15.71	-	7.42	864.56	23.35	84.37	41.47	42.90	1.03
H_2S	H2S-1	0.0	-2.0	-2.0	-2.0	-1.0	14.38	1.57	34.62	1400.80	18.16	85.57	23.07	62.50	2.71
	H2S-2	0.0	-1.0	-1.0	-2.0	-3.0	14.18	1.45	28.56	1910.19	10.62	86.76	46.08	40.68	0.88
	H2S-3	0.0	-1.5	-1.0	-1.0	-3.5	15.26	1.87	55.03	764.08	6.89	84.37	30.28	54.09	1.79
	H2S-4	0.0	-2.0	-1.0	-1.0	0.0	11.73	1.62	33.46	1528.15	7.80	85.97	23.25	62.72	2.70
	H2S-5	0.0	-1.5	-1.0	-2.0	0.0	12.61	-	35.85	1782.84	10.16	83.57	24.64	58.93	2.39
	H2S-6	0.0	0.0	-1.0	-1.0	-1.0	11.44	-	30.86	1589.68	24.07	82.35	27.78	54.57	1.96
	H2S-7	0.0	0.0	-1.0	-1.0	-1.0	-	-	-	-	-	-	-	-	-
	H2S-8	0.0	0.0	-1.0	-1.5	-1.5	-	-	-	-	-	-	-	-	-
	H2S-9	0.0	-1.0	-1.0	0.5	0.0	-	-	-	-	-	-	-	-	-
	H2S-10	0.0	0.0	-1.0	-1.0	-0.5	-	-	-	-	-	-	-	-	-
	H2S-11	0.0	-1.5	-2.0	-2.0	-2.0	-	-	-	-	-	-	-	-	-
	H2S-12	0.0	-1.0	-1.5	-2.0	-3.0	-	-	-	-	-	-	-	-	-
